# Amyloid Probability in Alzheimer Disease From Plasma, Cerebrospinal Fluid, and Amyloid Imaging

**DOI:** 10.21203/rs.3.rs-8492792/v1

**Published:** 2026-01-12

**Authors:** Khushboo Verma, Satwant Kumar

**Affiliations:** University of Texas at Tyler; Alzheimer’s Alliance of Smith County

## Abstract

Blood-based biomarkers are increasingly used to triage patients for amyloid confirmation, yet performance is often reported versus a single comparator despite discordance between amyloid PET and CSF. In a cross-sectional secondary analysis of the Alzheimer’s Disease Neuroimaging Initiative (ADNI), we assembled one PET-anchored PET–CSF–plasma triad per participant (n = 320). Bayesian latent class models integrating PET, CSF and plasma (Aβ42/40 or %p-tau217) estimated pattern-level posterior probabilities of latent amyloid positivity, with prespecified sensitivity analyses for PET–CSF conditional dependence, timing gaps (≤ 7, 8–30, > 30 days) and CSF cutpoints. Concordant PET+/CSF + and PET−/CSF − patterns mapped to probabilities near 1 and 0, whereas discordant patterns yielded intermediate probabilities refined by plasma strata and most sensitive to dependence assumptions. PET–CSF discordance occurred even within ≤ 7 days (12/98; 12%). A CSF Aβ42 coverage analysis showed similar gradients. Pattern-to-probability reporting may aid interpretation without privileging a reference test.

## Introduction

Determining whether an individual harbors Alzheimer-type brain amyloid has become increasingly important as biomarker confirmation now shapes diagnostic pathways and eligibility for amyloid-targeting therapeutic and research programs^1-3^. Blood-based biomarkers (BBMs) are emerging as scalable, low-burden gatekeepers that estimate amyloid likelihood and can triage patients for confirmatory testing, while current appropriate-use recommendations still emphasize confirmation with cerebrospinal fluid (CSF) measures and/or amyloid PET^2,3^. In parallel, BBMs are being incorporated into trial screening and enrichment workflows to reduce burden and screen failure, even as PET and CSF remain the predominant confirmatory modalities in many protocols^1-4^.

However, amyloid “confirmation” is not always unambiguous: amyloid PET and CSF amyloid measures, while generally concordant, can yield discordant classifications in a clinically meaningful minority of individuals (often ~ 10–20%)^5,6^. These discordant profiles are not merely technical noise; multiple longitudinal and biomarker studies suggest they can carry biological and prognostic information, including differential associations with downstream tau pathology and clinical trajectories depending on whether CSF becomes abnormal before PET or vice versa^7,8^. As a result, “PET + vs CSF+” is not a trivial labeling choice, discordance creates a real interpretive gap for both clinical decision-making and study design when the two most common confirmatory modalities disagree^5,8^.

Despite reported discordances diagnostic performance is still commonly framed “versus PET” or “versus CSF,” as if one modality were a definitive ground truth. This convention is increasingly strained: although both Aβ-PET and CSF amyloid assays aim to detect amyloid plaque pathology, they index different (but related) biological processes^9,10^, with PET tracking fibrillar plaque burden and CSF reflecting soluble Aβ dynamics^10^. Whereas a clinically meaningful minority of individuals show persistent PET–CSF discordance^7^. When plasma biomarkers are evaluated against a single comparator, discordant and borderline cases are forced into “false-positive/false-negative” bins by design choice rather than biology, and even very high-performing blood tests inherit the uncertainty (and potential ceiling) of an imperfect reference^11,12^. In practice, reported “accuracy” can therefore shift with the selected reference test and thresholds, while clinicians and trialists remain without a calibrated probability statement for the common real-world scenario of PET–CSF disagreement^4,12^.

A natural way to resolve this reference-standard problem is to treat amyloid status as a latent (unobserved) state and jointly model PET, CSF, and plasma as imperfect indicators of that state, rather than forcing one modality to serve as ground truth^13^. Latent class models were developed precisely for diagnostic settings without a single accurate reference standard and can yield directly interpretable outputs such as P *(amyloid+)* given the observed test pattern, with uncertainty^13,14^. Importantly, biomarker applications must also confront conditional dependence, for example, PET and CSF may remain correlated even after conditioning on latent amyloid. This assumption violation can materially bias inference if left implicit. To incorporate (and stress-test) such dependence structures Bayesian formulations provide a principled way^14,15^. In this context, adding plasma as a third modality is not only clinically pragmatic but also methodologically valuable: an additional indicator can refine posterior probabilities for discordant PET–CSF patterns and improve identifiability under plausible dependence scenarios^15,16^.

Any integrative approach in this setting must make its assumptions explicit. Latent class inference without a gold standard is identifiable only under modeling constraints, and results can be sensitive to the commonly made (and often violated) assumption that tests are conditionally independent given the latent state, particularly for PET and CSF, which share biology and measurement pipelines^14,15^. In addition, amyloid classifications depend on assay-specific cutpoints (especially for CSF), where small threshold shifts can move borderline individuals across the positivity boundary and thereby change apparent discordance rates^17^. Finally, because PET and CSF may not be acquired on the same day in observational cohorts, the time gap between modalities is a plausible contributor to disagreement and should be treated as a prespecified sensitivity axis rather than a post hoc explanation^6,8^. Together, these considerations argue for reporting calibrated, pattern-level probabilities with uncertainty and for stress-testing conclusions across dependence, timing, and cutpoint assumptions^14,15^.

Here, in a cross-sectional secondary analysis of the Alzheimer’s Disease Neuroimaging Initiative (ADNI)^18^, we assembled per-participant PET–CSF–plasma “triads” and used Bayesian latent class models to estimate the posterior probability of a latent amyloid-positive state for each observed PET–CSF–plasma pattern, rather than defining truth by a single modality. Using Elecsys CSF Aβ42/40 as the primary CSF amyloid definition and plasma measurements aligned to the PrecivityAD2 component analytes (plasma Aβ42/40 ratio or plasma %p-tau217), we quantify how plasma strata refine interpretation of discordant PET–CSF profiles^17,19-21^. We prespecified sensitivity analyses for PET–CSF conditional dependence, modality timing gaps, and CSF cutpoints, and we performed a coverage analysis using Elecsys CSF Aβ42 to address incomplete availability of CSF Aβ42/40^8,17^. Together, these analyses yield a clinically interpretable “pattern-to-probability” output with explicit uncertainty for common real-world discordant and borderline biomarker patterns.

## Results

### Cohort and analytic sets

**Figure 1:**
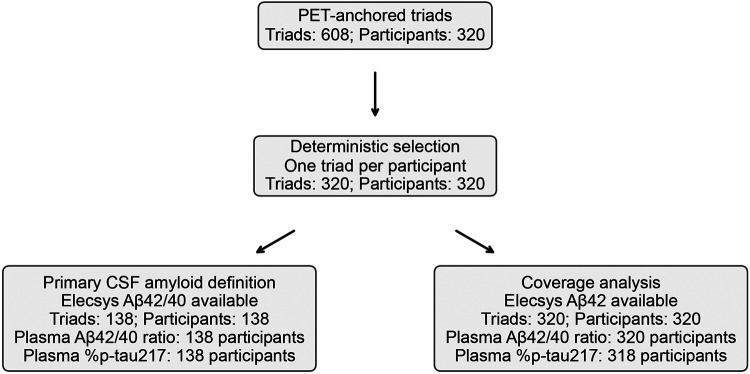
Cohort construction and analytic sets Flow diagram summarizing cohort construction from PET-anchored triads and the analytic sets used for the primary analysis (Elecsys CSF Aβ42/40 available) and the prespecified coverage analysis (Elecsys CSF Aβ42 available). Counts are shown as triads and unique participants (research identification numbers).

The PET-anchored cohort comprised 608 eligible PET–CSF–plasma triads from 320 participants; selecting one prespecified triad per participant yielded 320 triads for analysis (Fig. 1). Participants had a mean (s.d.) age of 71.1 (6.7) years and included 162 women (51%). Elecsys CSF Aβ42/40 was available for 138/320 participants (43%), defining the prespecified primary analysis set for both plasma endpoints (Fig. 1). Included and excluded participants were similar in age, sex, and APOE ε4 frequency, but differed in diagnostic composition: the included set contained a higher proportion of cognitively unimpaired individuals (84/138 [61%] vs 76/182 [42%]) and a lower proportion with mild cognitive impairment (38/138 [28%] vs 93/182 [51%]) (Table 1).

### Pattern-to-probability posteriors

**Figure 2:**
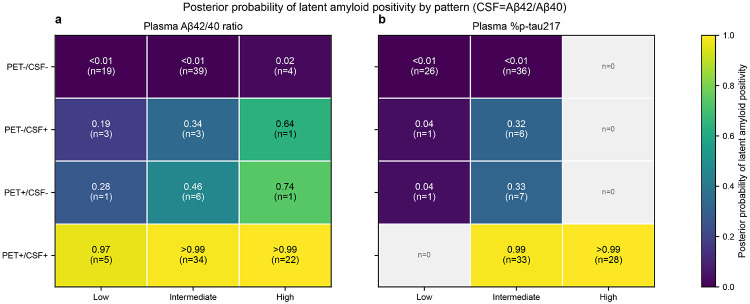
Posterior probability of latent amyloid positivity by biomarker pattern Heatmaps show the posterior mean probability of latent amyloid positivity (A*) for each observed PET– CSF–plasma pattern in the primary CSF Aβ42/40 analysis. a) Plasma Aβ42/40 ratio strata. b) Plasma %p-tau217 strata. Rows indicate PET and CSF amyloid status; columns indicate plasma strata (low/intermediate/high). Each cell shows the posterior mean and the observed cell count (n). Cells shaded light gray indicate unobserved patterns (n=0).

In the primary CSF Aβ42/40 analysis set, concordant PET+/CSF+ patterns yielded posterior probabilities of latent amyloid positivity near 1, whereas concordant PET−/CSF− patterns yielded probabilities near 0, across plasma strata for both plasma Aβ42/40 ratio and plasma %p-tau217 (Fig. 2; **Supplementary Data 1**). For plasma Aβ42/40 ratio, the estimated *P*(*A* = 1) was 0.998 (95% credible interval [CrI], 0.992–1.000) for PET+/CSF+/High (n=22) and 0.001 (95% CrI, <0.01) for PET−/CSF−/Low (n=19). In contrast, discordant PET–CSF patterns mapped to intermediate posterior probabilities with wider uncertainty, and were refined by plasma strata; for example, PET+/CSF−/Intermediate had P(A = 1) 0.462 (95% CrI, 0.025–0.961; n=6). Plasma quantile cutpoints used to define low/intermediate/high strata are provided in **Supplementary Data 2**.

For plasma %p-tau217, *P(A* = 1) was 0.9997 (95% CrI, 0.998–1.000) for PET+/CSF+/High (n=28) and 0.0001 (95% CrI, <0.001) for PET−/CSF−/Low (n=26) (**Supplementary Data 1**). Discordant patterns again produced intermediate probabilities; for example, PET+/CSF−/Intermediate had *P(A* = 1) 0.325 (95% CrI, 0.010–0.876; n=7) and PET−/CSF+/Intermediate had *P(A* = 1) 0.323 (95% CrI, 0.011–0.865; n=6). Several PET–CSF–plasma combinations were unobserved (n=0) (Fig. 2), reflecting sparse cells for some patterns.

#### Timing-stratified discordance

**Figure 3:**
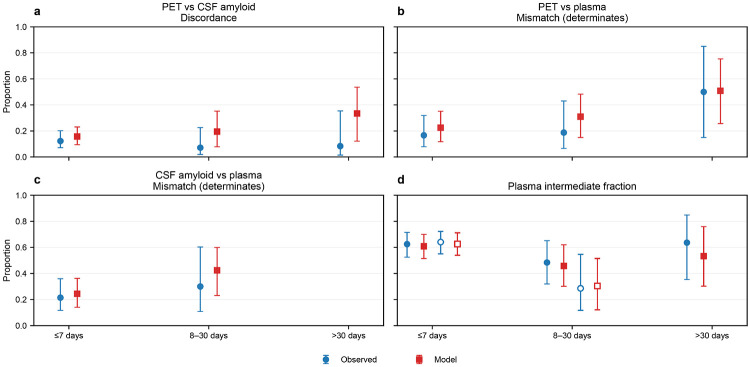
Timing-stratified discordance across PET, CSF, and plasma Aβ42/40 ratio Timing-stratified discordance and mismatch summaries for plasma Aβ42/40 ratio across prespecified absolute time-gap strata (≤7 days, 8–30 days, >30 days). a) PET vs CSF amyloid discordance by ∣ΔPET–CSF∣. b) PET vs plasma mismatch among determinate plasma strata (low vs high) by ∣ΔPET–plasma∣. c) CSF amyloid vs plasma mismatch among determinate strata by ∣ΔCSF–plasma∣. d) Plasma intermediate fraction shown for PET–plasma (filled markers) and CSF amyloid–plasma (open markers). Observed estimates are shown as blue circles with 95% Wilson confidence intervals; model estimates are shown as red squares with 95% posterior predictive credible intervals. Timing strata with fewer than 10 determinate observations are not plotted.

**Figure 4:**
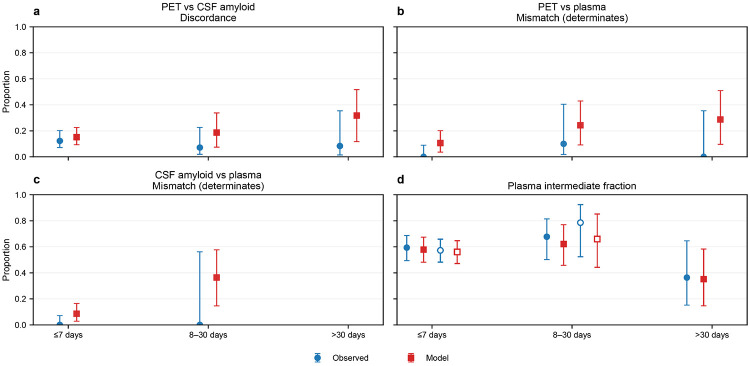
Timing-stratified discordance across PET, CSF, and plasma %p-tau217 Same format as Fig. 3, but with plasma strata defined by plasma %p-tau217. a) PET vs CSF amyloid discordance. b) PET vs plasma mismatch among determinate plasma strata (low vs high). c) CSF amyloid vs plasma mismatch among determinate strata. d) Plasma intermediate fraction shown for PET–plasma (filled markers) and CSF amyloid–plasma (open markers). Observed estimates are shown as blue circles with 95% Wilson confidence intervals; model estimates are shown as red squares with 95% posterior predictive credible intervals. Timing strata with fewer than 10 determinate observations are not plotted.

PET–CSF discordance was present across all prespecified PET–CSF timing strata (Figs. 3-4; **Supplementary Data 3**). In the ≤7-day stratum, 12/98 participants were discordant (12%; 95% CI, 7–20%). Discordance was 2/28 (7%; 95% CI, 2–23%) for 8–30 days and 1/12 (8%; 95% CI, 1–35%) for >30 days, with limited precision in longer-gap strata due to smaller denominators. Model-based posterior predictive PET–CSF discordance in the ≤7-day stratum was 0.16 (95% CrI, 0.09–0.23) for plasma Aβ42/40 ratio and 0.15 (95% CrI, 0.09–0.23) for plasma %p-tau217 (**Supplementary Data 4**).

Within the ≤7-day stratum, the intermediate plasma category represented the majority of observations in PET–plasma comparisons (60/96 [63%] for plasma Aβ42/40 ratio; 57/96 [59%] for plasma %p-tau217) (**Supplementary Data 3**). Restricting to determinate plasma strata (low vs high), mismatch with PET was 6/36 (17%) for plasma Aβ42/40 ratio and 0/39 (0%) for plasma %p-tau217. Similarly, in CSF–plasma comparisons within ≤7 days (n=117), intermediate plasma comprised 75/117 (64%) for plasma Aβ42/40 ratio and 67/117 (57%) for plasma %p-tau217; mismatch among determinate strata was 9/42 (21%) for plasma Aβ42/40 ratio and 0/50 (0%) for plasma %p-tau217. Estimates in longer timing strata were less precise due to smaller denominators (**Supplementary Data 3**).

### Sensitivity and coverage analyses

Median Centiloids and CSF Aβ42/40 values varied systematically across PET–CSF–plasma patterns, supporting biological coherence of the pattern-to-probability gradients (**Supplementary Fig. 1**; **Supplementary Data 6**). In prespecified sensitivity analyses, posterior probabilities for concordant PET+/CSF+ and PET−/CSF− patterns remained stably near 1 and near 0, respectively, whereas discordant and borderline patterns showed the greatest sensitivity to model assumptions—including PET–CSF conditional dependence, CSF cutpoint bands, and plasma discretization choices (**Supplementary Figs. 2–5**; **Supplementary Data 5**). Model convergence diagnostics are reported in **Supplementary Data 7**.

To address incomplete availability of CSF Aβ42/40, we performed a prespecified coverage analysis using Elecsys CSF Aβ42 (n=320). Pattern-level posterior probabilities showed similar gradients across plasma strata to the primary analysis (**Supplementary Fig. 6**; **Supplementary Data 8**). Observed PET–CSF discordance in the coverage cohort was ~0.19 for ≤7 days and ~0.15 for 8–30 days (**Supplementary Data 9**), with timing-stratified posterior predictive discordance estimates and timing decompositions shown in **Supplementary Data 10** and **Supplementary Figs. 7–8**. Coverage plasma quantile cutpoints and key sensitivity posteriors are provided in **Supplementary Data 11–12**.

## Discussion

In paired PET–CSF–plasma triads, Bayesian latent class modeling reframes amyloid assessment as estimation of a latent amyloid-positive state and yields directly interpretable, reference-standard–independent posterior probabilities for each observed biomarker pattern^13,14^. Within this framework, concordant PET+/CSF+ and PET−/CSF− profiles mapped to posteriors near 1 and near 0, respectively, whereas PET–CSF discordance, observed in a meaningful minority of individuals in prior cohorts, was mapped to intermediate probabilities that were systematically refined by plasma strata^1,5^. Together, these results support a clinically usable “pattern-to-probability” interpretation of amyloid biomarkers that avoids treating PET or CSF as ground truth in cases where they disagree^14^.

Two features of the analysis are particularly important for interpretation. First, allowing residual PET–CSF dependence materially affected posterior probabilities for some discordant and borderline patterns, reinforcing that conditional independence should not be assumed by default in no–gold-standard biomarker settings and is best treated as a prespecified sensitivity axis^14,15^. Second, PET–CSF discordance persisted even when modalities were closely paired in time, suggesting that disagreement is not simply a timing artifact within common acquisition windows; rather, it is consistent with evidence that PET–CSF discordance represents a typical transitional stage in amyloid biomarker evolution (with CSF-first and PET-first pathways)^5,6,10^. Nonetheless, inference about timing effects in longer-gap strata remains limited by sparse denominators, underscoring the need for larger, intentionally time-aligned datasets to isolate temporal contributions to discordance.

A central advantage of this framework is that it is explicitly comparator-agnostic. Rather than reporting agreement “vs PET” or “vs CSF”, metrics that are inherently reference-standard–dependent when the comparator itself is imperfect and PET–CSF discordance occurs in routine cohorts, we estimate P(amyloid — positive) for the observed PET–CSF–plasma pattern and report uncertainty around that estimate^13,14,22,23^. This is aligned with contemporary biomarker workflows in which blood tests support triage and, when needed, escalate to confirmatory testing, but do not fully eliminate ambiguity in intermediate or discordant cases^1,2,12^. Finally, because CSF Aβ42/40 ratios (which often improve concordance with PET relative to Aβ42 alone) are not universally available across datasets and platforms, we prespecified a CSF Aβ42–based coverage analysis to evaluate whether the pattern-to-probability gradients were preserved when extending to the full cohort while retaining CSF Aβ42/40 as the primary definition ^10,17^.

Several limitations merit emphasis. First, latent class inference in the absence of a gold standard is identifiable only under explicit modeling constraints (including assumptions about conditional dependence), and posterior probabilities for discordant and borderline patterns can therefore be sensitive to these specifications, even when concordant patterns remain stable^15,24,25^. Second, ADNI is a deeply phenotyped research cohort designed to approximate trial-like sampling, but its case-mix and limited ethnocultural diversity can constrain generalizability and may introduce spectrum effects when transporting pattern-level probabilities to routine clinical populations^25-27^. Third, we discretized continuous plasma markers into low/intermediate/high strata to improve interpretability and align with triage-style reporting; however, categorization can reduce information and precision relative to continuous modeling, and thresholds may not transfer across assays and populations^28,29^. Finally, some pattern cells and longer timing strata were sparse, limiting precision for timing-specific estimates and motivating replication in larger, deliberately time-aligned datasets.

These findings have practical implications for both clinical workflows and study reporting. As blood-based biomarkers are increasingly used to triage patients for confirmatory amyloid assessment, a pattern-to-probability output can provide a transparent way to communicate uncertainty^1,3^. This particularly applicable for the common “gray-zone” scenario of PET–CSF disagreement, while remaining compatible with appropriate-use recommendations that still emphasize PET and/or CSF confirmation in many contexts^2,8,9^. More broadly, our results motivate a shift from single-comparator accuracy summaries toward pattern-level probability reporting with prespecified sensitivity analyses, which can improve comparability across studies and reduce implicit overconfidence driven by reference-standard choice^2,12^. Future work should validate these pattern-level posteriors in external, more demographically diverse cohorts; evaluate continuous (rather than discretized) plasma models calibrated to workflow-aligned thresholds; and extend the framework to joint modeling of amyloid with tau and neurodegeneration markers and clinically relevant outcomes, enabling decision support that better matches the multi-modal reality of Alzheimer biomarker implementation^2,12^.

## Methods

### Study design and data source

This study was a cross-sectional secondary analysis of the Alzheimer’s Disease Neuroimaging Initiative (ADNI)^18^, a multicenter, longitudinal observational cohort. ADNI data were accessed through the ADNI data repository and included acquisitions collected between 2010 and 2022; all analyses were conducted in December 2025.

### Ethics approvals and consent

ADNI study procedures were approved by the institutional review board at each participating site, and all participants (or their legally authorized representatives) provided written informed consent. This secondary analysis used deidentified ADNI participant-level data accessed through the ADNI repository and involved no new participant recruitment or biospecimen collection. The University of Texas at Tyler Institutional Review Board determined this work to be Not Human Subjects Research (IRB #2025-286).

#### Participants and PET-anchored triad construction

The analytic unit was a PET-anchored triad consisting of (i) an amyloid PET scan, (ii) a CSF collection, and (iii) a plasma collection from the same participant. Triads were constructed using prespecified symmetric pairing windows of ±90 days for PET–plasma and ±180 days for PET–CSF and plasma–CSF. When multiple plasma and/or CSF observations were eligible for a given PET scan, we selected the PET–CSF–plasma combination that minimized, in order, (1) the maximum absolute pairwise time gap and then (2) the sum of absolute pairwise time gaps across PET–plasma, PET–CSF, and plasma–CSF; deterministic tie-breakers favored smaller gaps and earlier acquisition dates.

For the primary analysis, we selected one triad per participant (unique ADNI research identification number) to avoid within-participant correlation and to align the estimand with a cross-sectional probability of latent amyloid positivity at the PET anchor time. Triad selection was deterministic: we prioritized availability of the primary CSF Aβ42/40 measure and then minimized, in order, the maximum and sum of absolute pairwise day gaps. Analyses were performed as complete-case analyses for each plasma endpoint, excluding triads with missing PET status, CSF amyloid status, or the corresponding plasma endpoint.

### Biomarker measures and definitions

Amyloid PET status was defined using the ADNI UC Berkeley amyloid PET processing and summary measures, which provide a binary amyloid classification derived from standardized uptake value ratio (SUVR) summaries after PET–MRI co-registration and intensity normalization to a reference region^30^. For CSF, primary CSF amyloid positivity (CSF-A) was defined using the Roche Elecsys CSF Aβ42/40 ratio and a published ADNI Elecsys CSF-only (“algorithm”) cutpoint (0.0525)^17^. We also evaluated a prespecified multiplicative cutpoint band (0.90×, 0.95×, 1.00×, 1.05×, 1.10×) to quantify sensitivity of inferences to plausible CSF threshold variation^17^.

Because Elecsys CSF Aβ42/40 was available for only a subset of participants, we prespecified a coverage/transportability analysis that used Elecsys CSF Aβ42 alone to define CSF amyloid status in the full cohort (n=320), applying a published ADNI Elecsys CSF-only (“algorithm”) cutpoint (963 pg/mL) and the same multiplicative cutpoint band (0.90×–1.10×)^17,19^. Plasma biomarkers were derived from PrecivityAD2 component measurements obtained by high-throughput LC–MS/MS: plasma Aβ42/40 ratio and plasma %p-tau217^20,21^. For interpretability, each plasma endpoint was oriented so that higher values indicated greater likelihood of amyloid positivity and then discretized into low/intermediate/high strata using prespecified within-cohort quantiles (≤20th, 20th–80th, ≥80th percentile); a sensitivity analysis repeated key outputs using workflow-aligned triage thresholds^13,14,16,20^.

### Outcomes

The primary outcome was the pattern-level posterior probability of latent amyloid positivity at the PET anchor time, defined as

P(A=1∣PET status,CSF amyloid status,plasma stratum),

where *A* denotes the latent amyloid-positive state. Secondary outcomes quantified discordance (observed and posterior predictive) for PET–CSF (primary), and for PET–plasma and CSF–plasma (supporting). Timing strata were prespecified using absolute pairwise day gaps (≤7 days, 8–30 days, and >30 days up to 180 days) and applied separately to each pairwise comparison. For tri-category plasma strata, discordance summaries included the intermediate fraction and, among determinate strata (low vs high), the mismatch rate relative to the corresponding binary comparator.

### Statistics and reproducibility

We used Bayesian latent class models to integrate PET, CSF, and plasma as imperfect indicators of a latent amyloid state (*A*) without assuming any single modality as a gold standard^13,14,16^. In the baseline model, PET status, CSF amyloid status, and plasma stratum were assumed conditionally independent given *A*. Because PET and CSF may retain residual correlation even after conditioning on amyloid status, we prespecified a sensitivity model that allowed conditional dependence between PET and CSF by modeling their joint distribution within each latent class; plasma remained modeled as conditionally independent given *A*^14,15^.

Priors were prespecified and weakly informative: Beta(1,1) priors for latent prevalence and binary test parameters; Dirichlet(1,1,1) priors for plasma-category probabilities within each latent class; and, in the PET–CSF dependence sensitivity model, Dirichlet(1,1,1,1) priors for the within-class joint PET–CSF probabilities. Posterior sampling used Gibbs sampling with 4 chains (2,000 burn-in iterations; 5,000 retained draws per chain) for the primary and dependence models; timing-stratified models used 2 chains (1,500 burn-in; 3,000 retained draws). Convergence was assessed using split $\hat{R}$ and effective sample size diagnostics, and model fit was evaluated using posterior predictive checks comparing observed versus model-implied pattern frequencies and discordance rates^31-33^. We report posterior means with 95% credible intervals. For observed discordance proportions, we report 95% Wilson confidence intervals.

## Supplementary Material

This is a list of supplementary files associated with this preprint. Click to download.

- SupplementaryInformationV1SKKV.pdf
- SupplementaryData4TimingDiscordancePosteriorPredictivePrimaryCSFAbeta4240.csv
- SupplementaryData7ModelDiagnosticsPrimary.csv
- SupplementaryData9CoverageTimingDiscordanceObservedCSFAbeta42.csv
- SupplementaryData1PatternPosteriorTablePrimaryCSFAbeta4240.csv
- SupplementaryData6PlausibilityByPatternPrimary.csv
- SupplementaryData10CoverageTimingDiscordancePosteriorPredictiveCSFAbeta42.csv
- SupplementaryData8CoveragePatternPosteriorTableCSFAbeta42.csv
- SupplementaryData12CoverageSensitivityKeyPosteriors.csv
- SupplementaryData11CoveragePlasmaQuantileCutpoints.csv
- SupplementaryData2PlasmaQuantileCutpointsPrimary.csv
- SupplementaryData5SensitivityKeyPosteriorsPrimary.csv
- SupplementaryData3TimingDiscordanceObservedPrimaryCSFAbeta4240.csv

## Figures and Tables

**Table 1:** Representativeness (included vs excluded)

Group	No.	Age, mean (SD)	Female, No. (%)	APOE ε4+, No. (%)	CN, No. (%)	MCI, No. (%)	AD, No. (%)	Other/unknown, No. (%)
Included (CSF β-amyloid 42/40 available)	138	71.1 (7.1)	69 (50.0)	47 (34.1)	84 (60.9)	38 (27.5)	15 (10.9)	1 (0.7)
Excluded (CSF β-amyloid 42/40 unavailable)	182	71.2 (6.3)	93 (51.1)	60 (33.0)	76 (41.8)	93 (51.1)	8 (4.4)	5 (2.7)

Abbreviations: AD, Alzheimer disease dementia; APOE, apolipoprotein E; CN, cognitively unimpaired; CSF, cerebrospinal fluid; MCI, mild cognitive impairment.

## Data Availability

The data supporting this study are available from the Alzheimer’s Disease Neuroimaging Initiative (ADNI) and can be accessed through the ADNI data portal (ida.loni.usc.edu) subject to ADNI data-use agreements. Because these data were used under license, they are not redistributed with this manuscript. All analytic code required to reproduce the results is publicly available (see Code availability); qualified researchers can obtain the underlying ADNI datasets directly from ADNI following approval of a data access request. As such, the investigators within the ADNI contributed to the design and implementation of ADNI and/or provided data but did not participate in the analysis or writing of this report. A complete listing of ADNI investigators can be found at https://adni.loni.usc.edu/wp-content/uploads/how_to_apply/ADNI_Acknowledgement_List.pdf.

## Code availability

All code used to construct triads, fit models, and generate figures is available at: https://github.com/SatwantKumar/amyloid-probability-adni. To support reproducibility, the repository includes the full analysis workflow and figure scripts.
